# Elongation of Very Long-Chain Fatty Acids (ELOVL) in Atopic Dermatitis and the Cutaneous Adverse Effect AGEP of Drugs

**DOI:** 10.3390/ijms25179344

**Published:** 2024-08-28

**Authors:** Markus Blaess, René Csuk, Teresa Schätzl, Hans-Peter Deigner

**Affiliations:** 1 Institute of Precision Medicine, Medical and Life Sciences Faculty, Furtwangen University, Jakob-Kienzle-Str. 17, D-78054 Villingen-Schwenningen, Germany; 2 Organic Chemistry, Martin-Luther University Halle-Wittenberg, Kurt-Mothes, Str. 2, D-06120 Halle (Saale), Germany; 3 Fraunhofer Institute IZI, Leipzig, EXIM Department, Schillingallee 68, D-18057 Rostock, Germany; 4 Faculty of Science, Tuebingen University, Auf der Morgenstelle 8, D-72076 Tuebingen, Germany

**Keywords:** atopic dermatitis, fatty acid elongation, ELOVL, lysosome, cutaneous adverse effects, ceramide, basic therapy, lysosomotropism, AGEP, eczema

## Abstract

Atopic dermatitis (AD) is a common inflammatory skin disease, in particular among infants, and is characterized, among other things, by a modification in fatty acid and ceramide composition of the skin’s stratum corneum. Palmitic acid and stearic acid, along with C_16_-ceramide and 2-hydroxy C_16_-ceramide, occur strikingly in AD. They coincide with a simultaneous decrease in very long-chain ceramides and ultra-long-chain ceramides, which form the outermost lipid barrier. Ceramides originate from cellular sphingolipid/ceramide metabolism, comprising a well-orchestrated network of enzymes involving various ELOVLs and CerSs in the de novo ceramide synthesis and neutral and acid CERase in degradation. Contrasting changes in long-chain ceramides and very long-chain ceramides in AD can be more clearly explained by the compartmentalization of ceramide synthesis. According to our hypothesis, the origin of increased C_16_-ceramide and 2-hydroxy C_16_-ceramide is located in the lysosome. Conversely, the decreased ultra-long-chain and very long-chain ceramides are the result of impaired ELOVL fatty acid elongation. The suggested model’s key elements include the lysosomal aCERase, which has pH-dependent long-chain C_16_-ceramide synthase activity (revaCERase); the NADPH-activated step-in enzyme ELOVL6 for fatty acid elongation; and the coincidence of impaired ELOVL fatty acid elongation and an elevated lysosomal pH, which is considered to be the trigger for the altered ceramide biosynthesis in the lysosome. To maintain the ELOVL6 fatty acid elongation and the supply of NADPH and ATP to the cell, the polyunsaturated PPARG activator linoleic acid is considered to be one of the most suitable compounds. In the event that the increase in lysosomal pH is triggered by lysosomotropic compounds, compounds that disrupt the transmembrane proton gradient or force the breakdown of lysosomal proton pumps, non-HLA-classified AGEP may result.

## 1. Introduction

Atopic dermatitis (AD) is a prevalent inflammatory skin disorder with a chronic or relapsing disease course characterized by dry skin, erythema, lichenification, erosions, scaling, oozing/weeping, recurrent eczematous lesions, and intense pruritus affecting people of all ages and ethnicities, and having a substantial psychosocial impact on patients [1,2,3,4]. The hallmark of AD is a dysfunctional epidermal barrier, which is consistently observed in the skin of patients with AD, regardless of whether the skin is affected or unaffected. Reduced barrier function results in increased transepidermal water loss (TEWL) and pH, greater permeability, and decreased water retention in the stratum corneum [1,2,5]. Eczema and acute lesions in infants are characterized by poorly defined erythema with edema, vesicles, excoriations, and serous exudate, all of which can be widely distributed. In childhood, eczema becomes more localized and chronic than in infancy, with paler erythema, dry skin (xerosis), and poorly defined lesions often involving flexor surfaces with thickening (lichenification) of chronic areas. Adolescents and adults typically present with diffuse eczema, with lesions commonly affecting the hands, eyelids, and flexures [1]. The high prevalence of AD in industrialized countries, affecting up to 20% of children and 10% of adults [1,2], as well as the varying severity and sometimes unpredictable progression; the temporal, local, and age-related constraints on the use of topical corticosteroids; and the restriction in the strength of the applicable topical corticosteroids [2,6,7], pose a challenge in particular in pediatrics for the treatment of infants and babies.

Current topical therapies include various topical steroids as a first-line anti-inflammatory treatment, topical calcineurin inhibitors tacrolimus and pimecrolimus, as well as the more recently discovered phosphodiesterase 4 (PDE4) inhibitor crisaborole [3,6]. Fortunately, in recent years, the development of new topical and especially systemic therapeutics approaches has gained momentum, offering the hope of more treatment options in the future. A number of innovative topical small molecules are currently undergoing clinical trials which are designed with the aim of targeting the well-known phosphodiesterase 4 (PDE4) of the adaptive immune response or the new topical targets in the innate immune response such as the (Aryl-hydrocarbon-Receptor (AhR)), the sphingosine-1-phosphate receptor 1 (S1PR1), the tropomyosin receptor kinase (TRK), and the G protein-coupled receptor 19 (GPCR19) in the adaptive immune response, or the Janus kinase (JAK) [3]. Nevertheless, among the promising group of small molecule JAK-inhibitors, only abrocitinib has received marketing authorization for the treatment of AD in the EU (12 December 2021) [8]. In addition to novel small molecules, there is a growing trend towards the development of systemically active biologics (antibodies) targeting itching (anti-IL31, anti-IL36R), the innate immune response (anti-IL-33), and the adaptive immune response (anti-IL4a, anti-IL5a, anti-IL13, anti-IL17A, anti-IL22, and anti-IL23) [3].

Despite well their advanced clinical development, there is a paucity of data regarding the long-term (adverse) effects of the new drugs on the skin. According to currently available in-vivo knowledge, the severity of adverse effects is expected to be on a par with that of tacrolimus, pimecrolimus, and crisaborol, causing minor temporary mild-to-moderate pain/burning at the application site [9]. However, the well-known severe adverse effects of long-term topical steroid use, such as cheek telangiectasia, facial hypertrichosis, and skin atrophy [2,7,10], would be deemed unacceptable in new drugs. Due to their mechanism of action, some of these new drugs will be significantly delayed or even denied in receiving marketing authorization for their use in infants and adolescents.

All therapeutic strategies, whether well-established or in the development stage, share a common feature: the targeting of predominantly macromolecules, such as signaling proteins, their receptors, or enzymes involved in signal transduction. In contrast to the well-understood pathogenesis at the cellular level [3,11,12,13], the significant changes at the level of the keratinocyte metabolome, such as lipid and fatty acid composition [14,15,16,17], have received less attention so far. This review focuses on the putative underlying mechanisms for the altered lipid composition of affected skin areas observed in AD. The objective is to gain a deeper understanding of the existing alterations in lipids in the skin that is prone to or affected by AD in order to develop therapeutic and prophylactic approaches for AD that extend beyond biologicals, corticosteroids, or topical and systemic H_1_-antihistamines. H_1_-antihistamines have been employed for decades to relieve itch/pruritus in patients associated with eczema and AD. However, recent evidence suggests that they are ineffective, and the most recent amendment of the European guidelines recommends against their use [6].

A lipid metabolism-centered approach, targeted repurposing of approved drugs, and the skillful use of natural products may address changes in cutaneous lipid metabolism, improve AD therapy, and prevent the recurrence of mild and moderate eczema in affected and prone individuals. The concept discussed in this review is not limited to the explanation of the increase of long-chain ceramides and the fate of ultra-long-chain and very long-chain ceramides. Moreover, it facilitates the understanding of cutaneous drug adverse effects such as acute generalized exanthematous pustulosis (AGEP), polymorphic light eruption (PMLE/PLE), or the occurrence of Mallorca acne (acne aestivalis) at the level of cellular lipids as well as derived treatment options. This represents an advancement of the model of “compartmentalized ceramide metabolism in cells”, which was first introduced in 2019 [18]. Specifically, the model focuses on the elongation of very long-chain fatty acids and the lysosomal pH, which have been proposed to be prominent contributors to these skin conditions [19].

## 2. Ceramides, Ceramide Composition, and Epidermal Barrier (dys) Function

**Figure 1:**
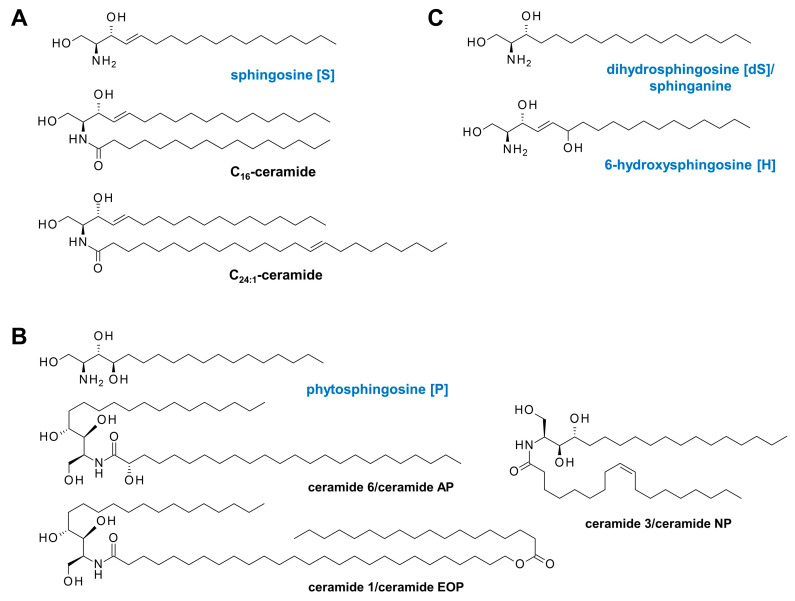
Backbones of various cutaneous ceramides, well-known ceramides in creams used to compensate cutaneous ceramide deficiency, and cell cycle relevant ceramides. (**A**) Cell cycle relevant ceramides C_16_-ceramide [NS] (pro-apoptotic, in AD strikingly increased) and C_24:1_-ceramide [NS] (cutaneous barrier essential) with sphingosine backbone [S] [14,20,22,23]. (**B**) Ceramide 1 (EOP), ceramide 3 (NP), and ceramide 6 (AP) with phytosphingosine [P] backbone listed in the INCI (International Nomenclature of Cosmetic Ingredients) list and commonly used in creams and lotions [24]. (**C**) Other relevant ceramide backbones: dihydroshingosine/sphinganine [dS] (e.g., in dihydroceramide) and 6-hydroxysphingosine [H].

Ceramides are well-characterized sphingolipid metabolites and second messengers in cells, consisting of a backbone of dihydrosphingosine (sphinganine) [dS], sphingosine [S], phytosphingosine [P], or 6-hydroxy-sphingosine [H]; and a fatty acid residue of non-hydroxy fatty acid [N], 2-hydroxy fatty acid [A], or esterified ω-hydroxy fatty acid [EO] (Figure 1) [5,16,20]. The typical chain length of the acyl moiety of cutaneous ceramides varies from C16 to C26; however, in the stratum corneum, the range present is up to C32 [11]. Depending on the carbon chain length of their fatty acid moiety, ceramides can be classified into three categories: long-chain ceramides (C_14_–C_18_/C_20_), very long-chain ceramides (C_20_–C_26_), and ultra-long-chain ceramides (>C_26_) [21]. As the ceramide classification is not yet standardized, some authors have classified them differently, categorizing them as short-chain ceramides (e.g., C_16_-ceramide), long-chain ceramides (e.g., C_24:1_-ceramide), and very long-chain ceramides (>C_26_) [5,14]. The subclass [NS], which includes the prominent pro-apoptotic C_16_-ceramide and the pro-proliferative and cell survival-inducing C_24:1_-ceramide, represents 7% of total dermal ceramide [15,20,22,23].

Within the sphingolipids, ceramides, in particular, exert a significant effect on the physicochemical characteristics of lipid membranes, including more complex structures such as the lipid barrier of the epidermis [22,25,26,27]. According to the current state of knowledge, the impact of ceramides on membrane characteristics depends predominantly on the structural features of the fatty acid moiety in particular. Saturated ceramides exert a more pronounced impact on the fluid membrane than their unsaturated counterparts, resulting in an increased order and promotion of gel/fluid phase separation. Very long-chain ceramides form tubular structures, possibly due to their capability to form interdigitated phases and dictate membrane lateral organization morphology [27]. Ceramides, whether present or released from precursors within membranes, can trigger an increased order of lipid chains, thereby facilitating the formation of the non-lamellar hexagonal II (HII) phase and the formation of microdomains (lipid rafts/caveolae). They promote membrane permeability and channel formation, induce flip-flop motion of lipids, facilitate membrane fusion, as well as induce fission, budding, vesicle/exosome formation, and exocytosis [22,25,26,28].

As highly versatile molecules, ceramides undergo intense metabolism, act as precursors of second messengers, form structural components in cells and membranes, or serve as second messengers, such as pro-apoptotic C_16_-ceramide or pro proliferative C_24_/C_24:1_-ceramide. They represent the endpoint of ceramide de novo synthesis, serving as a reactant for the storage form sphingomyelin (SM), the second messenger ceramide-1-phosphate (C1P), and the cerebrosides glucosylceramide (GluCer) and galactosyl-ceramide. Alternatively, they can be degraded to free fatty acid and sphingosine, the precursor of sphingosine-1-phosphate (S1P) [22,23,26,29,30,31]. A dysfunction in the precisely orchestrated network of enzymes is the most probable cause of the alterations in ceramide composition observed in non-lesional and lesional skin in AD [14].

## 3. Eczema and Atopic Dermatitis Pathogenesis on the Level of Ceramides and Fatty Acids

The stratum corneum is the outermost layer of the epidermis and is responsible for barrier function against transepidermal water loss (TEWL), consisting of distinct multi-lamellar membrane structures with a hydrophobic lipid mixture comprising mainly of free fatty acids, cholesterol, and ceramides [16,21]. Experimental data have demonstrated the crucial role of the proper composition of these components in maintaining the protective function of the epidermal barrier with a minimized TEWL. Minor modifications in the ceramide composition and increased TEWL are indicative of an incipient skin barrier dysfunction, which can be traced in the non-lesional skin of AD patients and significantly increased in the lesional skin of AD patients [5,32]. The composition of cutaneous ceramides affects lamellar arrangement and lipid organization, respectively, and subsequently the epidermal barrier function, TEWL, in the stratum corneum of AD patients and correlates with disease severity [5,14]. To date, the most prominent genetic risk factor for AD identified is associated with mutations in the skin barrier protein filaggrin, encoded by the FLG gene [12,33]. However, disease severity is independent of filaggrin mutations [5,14].

### 3.1. Modifications in (Very) Long-Chain Ceramides, (Very) Long-Chain Fatty Acids in AD

At the onset of AD, there is a change in the composition of lipids and fatty acids in both AD lesions and non-lesional skin. In non-lesional and lesional skin, the levels of the saturated fatty acids palmitic acid (C_16_) and stearic acid (C_18_) increase; however, the increase is more pronounced in lesions. In contrast to long-chain fatty acids, very long-chain fatty acids, particularly C_24_ fatty acids, exhibit the most decrease within lesions, while the ratio of saturated fatty acids to total fatty acids remains virtually unchanged. Substituted 2-hydroxy fatty acids do not exhibit this opposite effect, depending on the carbon chain length. In this instance, the level of long-chain 2-hydroxy palmitic acid remains virtually unaltered, whereas the levels of very long-chain 2-hydroxy fatty acids decrease more significantly in lesions compared to non-lesional skin [5,14]. In considering cutaneous ceramides, predominantly ceramides with 34 carbons (C34 ceramides) increase significantly within lesions, particularly the C_16_-ceramide (C34 ceramide, subclass [NS]) [5,14,17,34]. The C34-ceramides of the other subclasses [NdS], [NP], and [NH] also increase, although not to the same extent as the C_16_-ceramide [NS]. In the group of 2-hydroxy fatty acids-containing ceramides, only the C_16_ (2-hydroxy) ceramide [AS] increases significantly [14].

The increase in long-chain ceramides within skin lesions is accompanied by a corresponding decrease in ultra-long-chain and very long-chain ceramides that contain esterified ω-hydroxy fatty acids [EO]. The levels of very long-chain saturated and long-chain 2-hydroxy or ω-hydroxy fatty acids are significantly reduced in non-lesional skin and highly reduced in lesional skin. This is subsequently reflected in a decrease in ceramides belonging to the [EO] subclass. However, the extent of reduction varies depending on the type of sphingosine backbone. This reduction is more pronounced in the [EOS] and [EOH] subclasses compared to [EOP] and [EOdS] [14]. These findings are not unexpected, given that very long and ultra-long-chain non-hydroxy fatty acids [N] can be converted to esterified ω-hydroxy fatty acids by selective ω-hydroxylation of aliphatic hydrocarbon chains, followed by esterification with predominantly linoleic acid, and finally the formation of esterified ω-hydroxy fatty acids [EO] [29,35,36]. Consequently, in the absence of ultra-long-chain or very long-chain fatty acids in keratinocytes and the epidermis, less esterified ω-hydroxy fatty acids [EO] can be produced.

### 3.2. Pivotal Role of Ceramide and Fatty Acid Composition in AD

Given the altered composition (in ratio and amount) of ceramides and fatty acids in AD, it is reasonable to assume that the chain length and type of acyl moiety play a crucial role in determining the stratum corneum structure and the epidermal permeability of water and moisture (TEWL). Acyl moiety shortening of ceramides from very long-chain (≥C_24_) to long-chain (e.g., palmitic acid (C_16_-ceramide)) results in increased dermal moisture loss, particularly in a desiccating environment [5,15,37]. The impact is even more pronounced when ultra-long-chain fatty acids (≥C_28_) and ultra-long-chain ω-O-acyl-ceramides are barely present or absent, resulting in substantial morphological changes, including a histologically abnormal compacted outer epidermis (stratum corneum), deficient epidermal lamellar body content, and the absence of typical stratum corneum lamellar membranes [22,26]. These findings suggest that skin devoid of epidermal ω-O-acyl ceramides exhibits a deficiency in crucial hydrophobic components of the extracellular lamellar membranes in the stratum corneum, which serve to protect against epidermal water loss.

The altered composition, in ratio and amount, of ceramides in AD lesions, non-lesional skin, and skin prone to AD can be simulated and studied by in-vitro studies on prototype lipid membranes of the stratum corneum, which are composed of ceramides, free fatty acids, cholesterol, and sodium cholesteryl sulfate. The replacement of C_24_-ceramide [NS] with C_16_-ceramide [NS] demonstrated a significant impact on both microstructure and barrier function. Membranes with higher levels of C_16_-ceramide become significantly more permeable to water, resulting in higher TEWL [32] and an increased tendency towards drying the skin (xeroderma). This underscores the vital role of very long-chain ceramides in maintaining the barrier function of the stratum corneum. Consequently, maintaining a balance in both the ratio and the amount of the long-chain, very long-chain, and ultra-long-chain ceramide subtypes in the stratum corneum is essential for the resilience of the cutaneous barrier against TEWL.

## 4. Ceramide De Novo Synthesis Is a Multi-Step Process Affecting the Composition of Cellular and Cutaneous Ceramides

Ceramide de novo synthesis at the endoplasmatic reticulum (ER) of keratinocytes is a multistep process involving ceramide synthases (CerS) CerS3 and CerS4 [22,38], very long-chain-3-oxoacyl-CoA synthases (ELOVL), fatty acid synthase (FAS) [39], and long-chain-fatty-acid-CoA ligases (ASL) [40,41,42]. The activated acyl moieties in the penultimate step of the formation of the intermediate dihydroceramide by CerS can originate from multiple sources. These include the fundamental synthesis ending in C_16_-CoA and a subsequent elongation driven by ELOVL to long-chain (C_14_–C_18_/C_20_), very long-chain (C_20_–C_26_), or ultra-long-chain fatty acid-CoAs (>C_26_). Alternatively, the ATP-dependent (re)activation of pre-existing fatty acids from the hydrolysis of other lipids, such as triglycerides, phospholipids, or ceramides present in the lysosome, may occur. Each of the aforementioned steps can affect the ceramide composition in individuals with AD.

### 4.1. Activation of Free Fatty Acids—Conversion to Fatty Acid-CoA for the Elongation or Ceramide De Novo Synthesis

The activation of pre-existing free fatty acids occurs in a substrate-specific manner via various isoforms of the long-chain-fatty-acid-CoA ligase (ASL; EC 6.2.1.3.). Overall, thirteen isoforms have been yet identified, which are referred to as long-chain acyl-CoA synthases (ACSL1 and ACSL3–6) and very long-chain acyl-CoA synthases (ACSVL1–6) or fatty acid transport proteins (FATP1–FATP6), depending on the carbon chain length of the corresponding substrates [41,42,43]. Five ASCLs catalyze the conversion of fatty acids with a chain length of C_12_–C_20_ to their corresponding fatty acid-CoAs where ACSL1, ACSL5, and ACSL6 exhibit a preference for C_16:0_, whereas ACSL4 displays a preference for C_20:4_ [42]. In contrast, the ASCVLs/FATPs predominantly convert fatty acids with a carbon chain length of 20 or more (C_>20_) [40,41]. Each isoform is regulated independently and exhibits distinct tissue distribution, expression patterns, and subcellular locations [41].

The impairment of enzymes or mutations in enzymes involved in the activation of free fatty acids has been demonstrated to affect the skin structure and its homeostasis. In particular, the deletion of ACSVL5/FATP4 results in neonatal lethality in mice with a phenotype resembling ichthyosis prematurity syndrome, which is characterized by a skin covered at birth with a thick, caseous, desquamating epidermis changing to skin dryness, follicular hyperkeratosis, and fine white scaling at the scalp. This is sometimes associated with flexural dermatitis and atopic dermatitis [40,41,44,45]. Initially, it was proposed that ACSVL5/FATP4 plays a pivotal role in ceramide synthesis and skin homeostasis, particularly in the absence of ACSVL5/FATP4 activity in cells, which affects very long-chain ceramides [41]. However, studies on human fibroblasts did not support this hypothesis. In contrast to other molecules present in the skin, such as triacylglycerol, diacylglycerol, cholesterol esters, or phosphatidylcholine, each of which decreases by more than half, ceramides remain virtually unaltered [40]. These findings indicate that ACSVL5/FATP4 specifically affects the biosynthesis of other lipids, suggesting that a complex and uncompensated alteration of lipids other than ceramides is responsible for this type of severe skin dysfunction. Moreover, the altered ceramide profile observed in AD is independent of free fatty acid activation.

### 4.2. Elongation of Very Long-Chain Fatty Acids (ELOVL) Supplies the Acyl Moieties of Ceramides

The de novo biosynthesis of the very long-chain and ultra-long-chain fatty acid moieties (acyl-CoAs) of ceramides on other lipids is a subsequent two-step process that begins with the long-chain fatty acid synthesis (to C_16_-CoA) by FAS, or alternatively, with the aforementioned activation of fatty acids by ASL, which is followed by the elongation of the carbon chain of C_16_-, C_18_-, and C_26_-acyl-CoA by very long-chain-3-oxoacyl-CoA synthases (ELOVL1–7) at the ER. Of the seven ELOVLs, ELOVL1, ELOVL3, ELOVL4, ELOVL6, and ELOVL7 are involved in the elongation of saturated fatty acids and monounsaturated fatty acids [46,47,48]. The perfectly coordinated interaction between ELOVL1 (≥C_20_-acyl-CoA), ELOVL3 (especially to C_18_-acyl-CoA, but predominantly to C_22_-acyl-CoA), ELOVL6 (high affinity to C_16_-acyl-CoA and C_2_ chain extension of saturated fatty acids (from C_16_-acyl-CoA to C_18_-acyl-CoA)), and ELOVL4 (≥C_26_-acyl-CoA) are of particular interest in AD, as they enable the formation of a robust skin barrier [46,47,48].

Among the ELOVLs, a striking feature of ELOVL6 and ELOVL7 is the control of their enzymatic activity by NADPH, which increases the enzymatic activity of ELOVL6 by threefold and of ELOVL7 up to 10-fold without being a cofactor of either ELOVL. However, NADPH is a cofactor of 3-ketoacyl-CoA reductase, a component of the fatty acid elongation enzyme complex and involved in the downstream reduction step [49]. For the ELOVLs with minimal or no C_16_-acyl-CoA affinity, such as ELOVL1, ELOVL3, and particularly the vital ELOVL4, the initial elongation of C_16_-acyl-CoA to C_18_-acyl-CoA catalyzed by ELOVL6 is indispensable for the subsequent elongation.

### 4.3. ELOVL6 and ELOVL7 Are Clocks of ELOVL Elongation

The initial elongation of C_16_-acyl-CoA to C_18_-acyl-CoA is primarily carried out by ELOVL 6, providing the substrate for ELOVL3 and ELOVL1 for further elongation. Turnover rates vary depending on the NADPH concentration present [46,49]. In the event that both ELOVL6 and, depending on the tissue, ELOVL7 are either inactive or only minimally active, there is a heightened probability that free C_16_-acyl-CoA released from the FAS complex will be hydrolyzed to free palmitic acid by palmitoyl-CoA hydrolase (acyl-CoA hydrolase). A number of sources of oxidative stress that affect and decrease cellular NADPH levels and subsequently ELOVL6/ELOVL7 activity include UV radiation-induced reactive oxygen species (ROS), free radical/ROS inactivation, inadequate quenching of enzyme-derived ROS (e.g., amine oxidase (AO)), and photosensitizing xenobiotics in the epidermis [50,51,52,53]. Moreover, impairment of mitochondrial fatty acid oxidation impedes the vitality of the cell by decreasing the generation of FADH_2_, NADH, and ATP, thereby exacerbating oxidative stress [54]. For C_18_-acyl-CoA, a similar scenario of hydrolysis by the acyl-CoA hydrolase to stearic acid is possible, and indeed, an increase in free stearic acid can be detected in human skin with AD. However, the increase in C_18_-ceramide is somewhat less pronounced [14]. These findings are supported by observations in murine skin with ELOVL4-depletion, which results in a deficit of ultra-long-chain fatty acids and ceramides [26]. The importance of released free stearic acid and free palmitic acid will be discussed later, particularly in the lysosome under specific conditions.

### 4.4. Conversion of Ultra- and Very Long-Chain Fatty Acid-CoAs to Ceramides

Ceramide de novo synthesis at the ER represents the final step of this multistep process involving the six mammal ceramide synthases CerS1–CerS6 to form long-chain to ultra-long-chain ceramides from corresponding fatty acid-CoAs and a sphingosine backbone. In human keratinocytes, CerS3 and CerS4 are the most abundant, while CerS2, CerS5, and CerS6 are less abundant. Each of the enzymes displays characteristic substrate specificity towards acyl-CoAs [22,38]. The most abundant CerS3 in skin converts predominantly saturated C_18_- to C_26_-acyl-CoA to (very) long-chain ceramides, but also C_≥26_-acyl-CoA to ultra-long-chain ceramides. CerS4, which is significantly less abundant than CerS3 and preferentially converts saturated C_18_-acyl-CoA and C_20_-acyl-CoA, as well as C_22_-acyl-CoA and C_24_-acyl-CoA. In contrast, the rare CerS5 and CerS6 prefer C_16_-acyl-CoA [38,55].

## 5. Pivotal Role of Ultra- and Very Long-Chain Fatty Acid Elongation in Cells and the Skin

The maintenance of healthy skin and a tight skin barrier necessitates precise coordination between cutaneous ELOVLs and CerSs during the synthesis of essential fatty acids and ceramides. The substrate specificity of CerSs creates a dependence of ceramide composition on the presence and activity of specific ELOVLs in the corresponding tissue. In the absence of a properly functioning ELOVL4, CerS3, in particular, is unable to form ultra-long-chain ceramides, which are crucial for a robust skin barrier [21].

**Figure 2:**
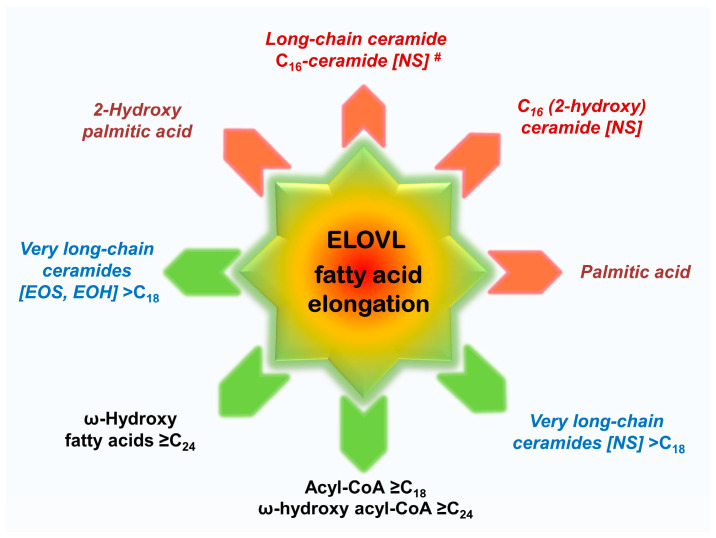
The pivotal role of ELOVL fatty acid elongation in cells and skin homeostasis. ELOVL fatty acid elongation is a source of essential very long-chain and ultra-long-chain fatty acids and the resulting cutaneous ceramides. Long-chain fatty acids and ceramides that are more abundant in the inflammatory process are colored red/dark red (orange arrows), while fatty acids and ceramides that are important for a stable skin barrier are colored blue/black (green arrows). (^#^) C_16_-ceramide is strikingly increased in AD, in skin prone to AD, and apoptosis [14,23].

The impact of ELOVL fatty acid elongation extends beyond the composition of ultra-long-chain, very long-chain, and long-chain fatty acids in cells to several other metabolic processes involving fatty acids. These processes include, but are not limited to, the de novo synthesis of ultra-long-chain, very long-chain, and long-chain ceramides with non-hydroxy fatty acid [N]; the biosynthesis of phosphatidyl glycerol, cardiolipin, cerebrosides, or diacylglycerol; fatty acids-related metabolism; ω-O-fatty acid esterification; and the lipoxygenase (LOX) pathway, among others (Figure 2). Of particular interest within the skin are fatty acid moieties present in triglycerides, phospholipids, and glycosylceramide [56].

### 5.1. ELOVLs Affect Chain Length of Fatty Acids, Fatty Acid Moiety of Ceramides, and Other Lipids

ELOVL-driven elongation, starting from C_16_-acyl-CoA, represents a pivotal metabolic pathway for cells, yielding a number of important compounds or precursors thereof. C_24_-ceramide and C_24:1_-ceramide are vital for proliferation, cell survival, and skin homeostasis [5,15,26,49], while ultra-long-chain ceramides and ultra-long-chain fatty acids are essential for protecting the organism against desiccation (Figure 2) [22,23,26]. As long as the elongation of ELOVL fatty acid is ongoing (indicated by green arrows in Figure 2), essential very long-chain and ultra-long-chain fatty acids and subsequently very long-chain ceramides (blue and black compounds) and ultra-long-chain ceramides (not shown) are formed. A decrease in the activity and turnover of the priming enzymes ELOVL6 and ELOV7, caused by NADPH depletion or enzyme malfunction of, e.g., ELOVL4 or ELOVL1 (red arrows in Figure 2), results in reduced levels of very long-chain and ultra-long-chain fatty acids and ceramides, respectively. These are replaced by the long-chain fatty acids, including palmitic acid and 2-hydroxy palmitic acid (red/dark red), as well as long-chain C_16_-ceramide and 2-hydroxy C_16_-ceramide which are considered to be linked to various skin dysfunctions (abnormal skin histology, increased TEWL), premature apoptosis, and cell fate [5,23,26].

### 5.2. Effects of Missing ELOVLs: Depletion of ELOVL 2 and ELOVL4 Causes Severe Skin Disorders

Keratinocytes lacking ultra-long-chain ceramides, as observed in ELOVL4-depleted mice, exhibit severe histologic abnormalities of the skin and dermal appendages, including scaling, xerosis, and premature death [26]. At the level of ceramides, the elimination of ELOVL4 results in an increase in long-chain ceramides, including C_16_-ceramide, 2-hydroxy C_16_-ceramide, C_18_-ceramide, 2-hydroxy C_18_-ceramide, and in particular very long-chain C_26_-ceramide and 2-hydroxy C_26_-ceramide, which represent the final products of ELOVL1 and the preferred substrates of ELOVL4. Consequently, the levels of ultra-long-chain fatty acids and ceramides (>C_26_) are substantially reduced [26]. The malfunction of ELOVL1 directly affects C_24_-fatty acids, C_24_-ceramides, and the derived lipids, which are essential for the composition and structure of the cell membrane. ELOVL1 catalyzes the formation of C_24_-acyl-CoA and C_24:1_-acyl-CoA, which are in turn converted to C_24_-ceramide and C_24:1_-ceramide. Both ceramides are subsequently transformed to sphingomyelin SM_24:0_ and SM_24:1_ by sphingomyelin synthases SMS1 and SMD2, which are located at the Golgi apparatus (SMS1) and in the plasma membrane (SMD2) [57]. A deficiency or dysfunction of ELOVL1 results in depletion of, for example, C_24_-ceramide, which ultimately leads to an increased transepidermal water loss (TEWL) [5,14,32] and an altered cell membrane composition, including a disrupted sphingomyelin-ceramide cycle, lipid rafts, and microdomains/ceramide-rich domains in the cell membrane [22,30]. This shift in fatty acid chain length affects the ceramide-derived ceramide-1-phosphat, glucosylceramide [26], and galactosylceramide, as well as other lipids such as phosphatidylcholine, lysophosphatidyl choline, triglycerides and phosphatidylethanolamine, to name but a few.

### 5.3. ELOVL3—A Bypass in NADPH Depletion for Ultra-Long and Very Long-Chain Fatty Acid Elongation

In addition to the predominant affinity of C_16_-acyl-CoA to ELOVL6, there is also an approximately half as strong affinity to ELOVL3, which, in contrast to ELOVL6 and ELOVL7, is NADPH independent [46,47,48]. In the event of a NADPH depletion, this characteristic enables keratinocytes to partially compensate for the reduced C_18_-acyl-CoA production by ELOVL6 and also prevents the availability of excessive C_16_-acyl-CoA for hydrolysis by palmitoyl-CoA hydrolase, resulting in the formation of free palmitic acid. The evidence for an ELOVL3-dependent compensatory mechanism is provided by the finding that the transcription (mRNA) of ELOVL3 was increased by more than 2.5-fold in the restored epidermis of psoriatic patients compared to the never-lesioned epidermis [58]. In non-lesional atopic eczema, the compensatory mechanism involving ELOVL3 appears to be sufficient, in contrast to AD lesions, where neither palmitic acid nor stearic acid increases can be blocked [14], suggesting partial or limited compensation. Other potential sources of free palmitic and stearic acid, such as (lyso-)phosphatidylcholine, phosphatidylethanolamine, or other lipids containing stearic acid, cannot be entirely excluded [59].

## 6. The Unknown Origin of Long-Chain C_16_- and C_18_-Ceramides in AD Lesional Skin

A common hypothesis frequently proposed to explain the increase in ceramides during apoptosis is the activation of ceramide de novo synthesis [60,61]. This hypothesis originated in the late 1990s when the radioactive diacylglycerol assay was predominantly used to quantify total ceramide. At that time, only a few ceramide quantification methods were available, most of them using radioactive ^32^P ATP and lacking sensitive quantification of individual ceramides [62,63]. Of the six mammalian CerS, CerS1, CerS5, and CerS6 have been identified as preferring C_16_-acetyl CoA and are, therefore, candidates for forming C_16_-ceramide within cells. Among them, CerS5 and CerS6 are present in the skin; however, their expression is relatively weak compared to Cers3 and Cers4 [38,48]. The extent to which the CerS are involved in the formation of ceramides, and in particular C_16_-ceramide, can be investigated with in vitro experiments using the CerS inhibitor fumonisin B1 [60]. During apoptosis, total ceramide levels increase, with only a portion of this increase prevented by fumonisin B1 [60]. However, the portion that can be inhibited does not include C_16_-ceramide, as C_16_-ceramide is increased during apoptosis in response to ionizing radiation [23]. In contrast, compounds with lysosomotropic characteristics, such as NB06, can prevent the increase in C_16_-ceramide induced by oxidative stress (minimally modified LDL (mmLDL), TNFalpha) and simultaneously enrich C_24:1_-ceramide under normal conditions, suggesting a pivotal role of the lysosome [64]. Consequently, it appears unlikely that CerSs are responsible for the increase in C_16_-ceramide. The question of the origin of C_16_-ceramide in lesional skin remains unanswered to date. A clear indication that the lysosome and its pH are likely to play an important role in the hypothesized selective synthesis is provided by the effect of lysosmotropic compounds on C_16_-ceramide upon induced oxidative stress [64,65].

The observed increase in ceramides containing palmitic acid and 2-hydroxy palmitic acid [5,14,23] suggests a highly specific synthesis rather than hydrolysis, given that no substrate specificity has been reported for the lysosomal hydrolases involved in ceramide metabolism, including acid sphingomyelinase (aSMase), glucosylceramidase, and galactoylsceramidase [64,66,67]. Moreover, membrane-bound neutral sphingomyelinase (nSMase) lacks substrate specificity [68], resulting in the release of individual ceramides by hydrolases in proportions consistent with their present in either their precursor or their storage form (e.g., sphingomyelin). No significant difference in sphingomyelin SM_16:0_ was found in the skin of AD patients compared to healthy controls [17]. Furthermore, in an unimpaired lysosome, released ceramide is immediately hydrolyzed to sphingosine and free fatty acid, inhibiting any detectable increase except a perhaps short-term increase [60]. Thus, the question of how the specific increase in C_16_-ceramide occurs remains unanswered. Our hypothesis of the compartmentalization of ceramide synthesis, the interaction of (impaired) ELOLVs with lysosomal pH, and the effect of pH on lysosomal acid ceramidase (aCERase) may provide an explanation for the specific increase in C_16_-ceramide.

## 7. Lysosomal pH, pH-Dependent Enzymatic Activity of aCERase, and Its Implication in AD

### 7.1. Lysosomal Proton Gradient and Its Implication in AD

The acidic physiological pH of (early) lysosomes (4.6–4.8) is predominantly generated and maintained by the vacuolar-type H^+^-ATPase (V-ATPase) and the lysosomal redox chain, another proton pump that transports protons into the lumen of lysosomes. The V-ATPase uses ATP as an energy source, whereas the lysosomal redox chain utilizes NADH from the cytoplasm [69,70]. Depletion of intracellular ATP or NADH levels leads to an increase in lysosomal pH, which affects the activity of lysosomal enzymes, including aSMase and aCERase. Typically, the decline in ATP or NADH levels is reversible when sufficient ATP or NADH is made available. The V-ATPase and the lysosomal redox chain differ in that the V-ATPase can be irreversibly blocked when ATP and NADH, as well as glutathione (GSH), are severely depleted during apoptosis and even more so during necrosis [71]. The catalytically active subunit of V-ATPase (73 kDa) contains a conserved region (P-Loop) with two cysteines at positions 254 and 532. These cysteines are capable of forming disulfide bonds that can lead to the inactivation of the active site of V-ATPase. Since the thiol-disulfide equilibrium is linked to the redox potential of the cytoplasm [69], a severe decrease in the cytosolic redox potential leads to the formation of the disulfide bond between Cys 254 and 532 and blocks the catalytically active ATPase site. Once formed, the disulfide bond is stable and can withstand physiological conditions. This is because the physiological concentration of GSH in the cytoplasm is insufficient to cleave the disulfide bond between the two cysteines and recover full V-ATPase activity [72]. The formation of this particular disulfide bond may be a point of no return in keratinocytes and a milestone in the development of skin lesions.

The determination of changes in lysosomal pH in lesional and non-lesional skin prone to AD compared to healthy skin can only be indirectly determined using the enzyme activity in cell lysates of cutaneous cells, as well-established methods using fluorescent probe molecules are not suitable [73,74]. Measurements of epidermal samples revealed that aSMase activity was lower in both lesional and non-lesional skin and correlated with an increase in TEWL [75]. This, in conjunction with an equally diminished lysosomal acid β-glucocerebrosidase activity [76], indicates an elevated lysosomal pH.

### 7.2. pH-Dependent Enzymatic Activities of aCERase

All currently known enzymes involved in the lysosomal metabolism of ceramide are either involved in the synthesis or degradation of ceramide or its derivatives. Their activity is pH-dependent. Of these enzymes, only the lysosomal aCERase exhibits a pH-dependent switch from a ceramide hydrolase to a ceramide synthase [77,78]. As a hydrolase, the aCERase has an optimum pH of 4.0–5.0, converting ceramides to sphingosine and a free fatty acid. With increasing pH, the ceramide hydrolase shifts to an unknown ceramide synthase (optimum of pH 5.5–6.5), the reverse acid ceramide synthase (revaCERase). The former hydrolase aCERase then joins the cellular ceramide synthases as an exceptional C_16_-ceramide and C_18_-ceramide synthesizing enzyme (revaCERase), which is atypically located in the lysosome instead of the ER [78]. RevaCERase exhibits a preference for reacting palmitic acid (C_16_) and stearic acid (C_18_) with sphingosine; however, exhibiting hardly any selectivity between palmitic acid and stearic acid [77,78]. In contrast to CerSs, the ATP-dependent activation of fatty acid moieties to acyl-CoA by ASL is not a prerequisite for the reaction to proceed [38,55,78]. The resynthesis of long-chain ceramides to form either C_16_-ceramide, C_18_-ceramide, or both is contingent upon the presence of the corresponding fatty acid within the lysosome. To observe a significant increase in C_16_-ceramide and 2-hydroxy C_16_-ceramide in lesional skin, a predominance of free palmitic acid and free 2-hydroxypalmitic acid is required. An increase in cellular pH and the loss of the aCERase hydrolase activity are accompanied by a non-specific accumulation of all ceramides, including those originating from ceramide de novo synthesis and the onset of the selective synthesis of C_16_-ceramide, resulting in a selective increase of C_16_-ceramide, particularly in the presence of free palmitic acid. This selective accumulation has been observed in apoptotic cells or in lesional skin [5,14,17,23].

## 8. The Compartmentalized Ceramide Synthesis Model Can Reproduce the Synthesis of C_16_-Ceramid/2-Hydroxy C_16_-Ceramid in Lesional Skin of AD

**Figure 3:**
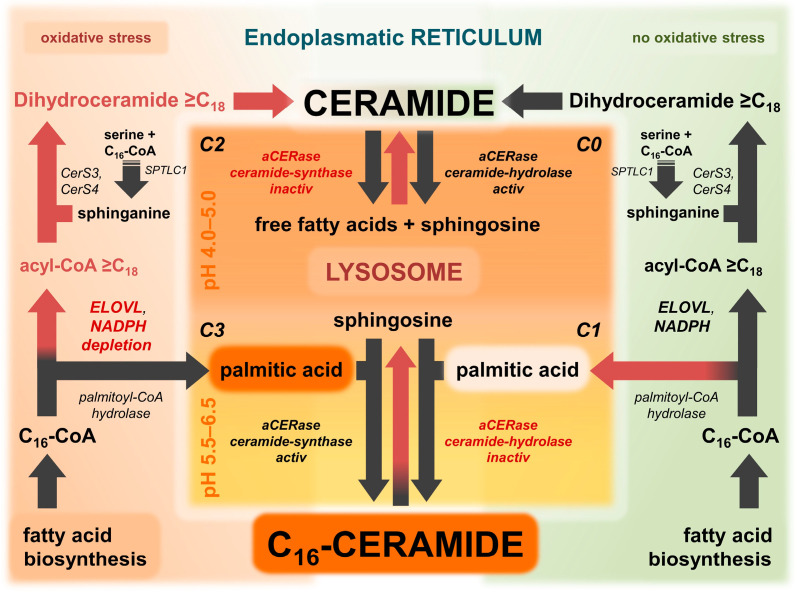
Lysosomal ceramide metabolism: an interplay of ELOVL6 activity, ELOVL fatty acid elongation, and lysosomal pH. In the compartmentalized model of ceramide metabolism, the synthesis of pro-apoptotic and in AD strikingly increased C_16_-ceramide is dependent on two factors: (a) lysosomal pH (type of aCERase activity) and (b) availability of NADPH (activity of ELOVL6). C_16_-ceramide is specifically formed upon NADPH depletion, subsequent termination of ELOVL6-initiated fatty acid elongation, and simultaneous increase in lysosomal pH above 5.5, which marks the transition of aCERase from ceramide hydrolase to lysosomal ceramide synthase (revaCERase) [77,78]. Black arrows indicate active pathways, while red arrows indicate inactive pathways. Possible combinations of ELOVL6 activity and lysosomal pH (conditions C0–C3) as indicated in Table 1 (condition 0–3): C0 (standard condition 0)—lysosomal pH normal and ELOVL6 elongation active; C1 (condition 1)—lysosomal pH elevated and ELOVL6 elongation active; C2 (condition 2)—lysosomal pH normal and ELOVL6 elongation impaired; C3 (condition 3)—lysosomal pH elevated and ELOVL6 elongation impaired. In the event that ELOVL6-initiated fatty acid elongation is not terminated, the accumulation of ultra-long-chain and very long-chain ceramides will occur in lysosomes at pH above 5.5 (condition 1).

Ceramide is involved in a complex network of enzymatic reactions that take place in different compartments of the cell (Figure 3). The de novo synthesis of ceramide is located at the ER, whereas the conversion to sphingomyelin by SMS1 and the glycosylation of ceramide catalyzed by GlcCer-synthase are located at the Golgi apparatus [57,67]. The release of ceramide from glucosylceramide or from sphingomyelin occurs in the lysosome. However, when ceramide serves as a signaling molecule, it is released from the precursor sphingomyelin by the membrane-bound nSMase [61,68,79]. The degradation to sphingosine occurs in various cellular compartments and is performed by pH-dependent ceramidases. In part, this degradation is carried out by neutral ceramidase (nCERase) in the early endosome, the precursor of lysosomes. However, the majority occurs in the late endosome/lysosome by aCERase [77,78,80].

### 8.1. ELOVL6 Activity and Lysosomal pH Control Lysosomal Synthesis and Degradation of Ceramide

Two regulatory factors derived from the compartmentalized model are the lysosomal pH and the ELOVL6-initiated very long-chain fatty acid elongation, which can operate lysosomal ceramide metabolism and ceramide levels and composition in cells. Given that the activity of ELOVL6 affects the initial step of very long-chain fatty acid elongation and thus the amount of free palmitic acid available, the suggested compartmentalization of ceramide synthesis results in different combinations of ELOVL6 activity and lysosomal pH, as illustrated in Figure 3 and Table 1. Three combinations that differ from standard conditions are of particular interest: (a) elevated lysosomal pH (5.5–6.5) and active ELOVL6 elongation, (b) normal lysosomal pH (4.0–5.0) and impaired ELOVL6 elongation, and (c) elevated lysosomal pH (5.5–6.5) and impaired ELOVL6 elongation (Figure 3). The following section will discuss and evaluate the four different scenarios without the presence of lysosmotropic compounds given by the model (Table 1) in order to assess their accuracy in predicting ceramide composition in, for example, AD. Prior to this, it is not necessary to know exactly what triggers the aCERase shift. For simplicity, only the predominant activity of aCERase is considered to be active. However, depending on the pH, various combinations of hydrolase and synthase activity of aCERase are present in lysosomes.

### 8.2. Condition 0: Lysosomal pH Normal and ELOVL6 Elongation Active

In physiological conditions (lysosomal pH 4.0–5.0 and the absence of oxidative stress/no depletion of NAD(P)H and ATP), ultra-long and very long-chain ceramides are synthesized predominantly by ceramide de novo synthesis (black arrows in Figure 3). The protection of cells from the critical accumulation of free C_16_-ceramide under physiological conditions appears to necessitate nCERase, which predominantly converts C_16_-ceramide to sphingosine and palmitic acid [79]. Sphingomyelin and ceramides are transported from the cell membrane along the endosomal pathway in the membrane of vesicles to the lysosome, where they are subsequently degraded by aCERase. In the depicted conditions (illustrated by red arrows in Figure 3), there is no lysosomal synthesis of long-chain ceramides.

### 8.3. Condition 1: Lysosomal pH Elevated and ELOVL6 Elongation Active

In experimental settings, lysosomotropic compounds such as NB06 can induce an elevated lysosomal pH in cells, resulting in the activation of the revaCERase activity of aCERase [61]. However, significant quantities of free palmitic acid are lacking to synthesize C_16_-ceramide. The formation of small amounts of C_16_-ceramide that are detectable is probably due to lysosomal phospholipase A2 (LPLA2) [61]. LPLA2 retains some residual activity for the hydrolysis of phosphatidylcholine at pH 5.0 to 6.5, which is capable of releasing palmitic acid [82] for the synthesis of C_16_-ceramide. In these conditions, other lysosomal enzymes such as aSMase, glycosylceramidase [83], and lysosomal acid lipase (LAL) [84], are virtually inactive and ultra-long-chain and very long-chain ceramides accumulate due to the lack of aCERase activity, This accumulation has been demonstrated in vitro with the lysosomotropic compound NB06 [61].

### 8.4. Condition 2: Lysosomal pH Normal and ELOVL6 Elongation Impaired

During oxidative stress, the ELOVL6 fatty acid elongation is impaired, resulting in an increased availability of palmitic acid due to hydrolysis of C_16_-CoA by palmitoyl-CoA hydrolase. Given the virtually constant pH in the lysosome, the revaCERase activity that is required to produce significant amounts of C_16_-ceramide is relatively low compared to the predominant aCERase activity. Nevertheless, a mild increase in C_16_-ceramide has been observed in non-lesional skin [14,17], which may be attributed to conditions of moderately elevated pH and partially impaired ELOVL6 fatty acid elongation. The previously discussed phenomenon of ELOVL6 impairment being bypassed by ELOVL3 in order to initiate fatty acid elongation seems to be capable of maintaining ultra-long-chain and very long-chain ceramides at approximately normal levels, as observed in non-lesional skin [5,17]. In these conditions, it is unlikely that significant increases in ceramides will be observed.

### 8.5. Condition 3: Lysosomal pH Elevated and ELOVL6 Elongation Impaired

The simultaneous increase of lysosomal pH and impairment of ELOVL6 elongation provide the ideal conditions for revaCERase to selectively synthesize C_16_-ceramide in the lysosome of, e.g., keratinocytes (Figure 3). Severe oxidative stress (lack of NAD(P)H, ATP, and GSH) in cells affects both the lysosomal proton pumps and the NADPH-dependent ELOVL6. The lack of NADPH impairs the initial conversion of C_16_-acyl-CoA to C_18_-acyl-CoA by ELOVL6, thereby inhibiting the synthesis of very long-chain-acyl-CoA. Conversely, the hydrolysis of C_16_-acyl-CoA to free palmitic acid is increased, resulting in subsequent conversion with sphingosine to C_16_-ceramide by revaCERase, which is now at its pH optimum (Figure 3). As observed in AD lesions [5,17], the result is a significant increase in C_16_-ceramide and a concomitant decrease in ultra-long-chain and very long-chain ceramides. With the elevated lysosomal pH, there is an inhibition in aCERase activity, which in turn results in the predominant accumulation of the synthesized C_16_-ceramide, as well as other ceramides from multiple sources that are no longer hydrolyzed within the lysosome. When 2-hydroxypalmitic acid is reacted with sphingosine instead of palmitic acid by revaCERase, the result is 2-hydroxy C_16_-ceramide, which is also significantly increased in AD [5]. However, at present, there is still a lack of studies on the substrate specificity of revaCERase with unbranched 2-hydroxy fatty acids that support this hypothesis.

## 9. (Severe) Cutaneous Adverse Reaction AGEP, Lysosomotropism of Drugs, and ELOVL6 Initiated Very Long-Chain Fatty Acid Elongation

Undesirable adverse effects or hypersensitivity reactions are generally considered to be mediated by B-cells (e.g., urticaria) and T-cells (e.g., eczema) [85,86,87]. However, small molecules, such as most drugs, are usually recognized directly only by cellular or soluble immunoglobulins (immediate and IgE-mediated reactions) rather than by immunocompetent B-cells and T-cells. Small, chemically reactive molecules (haptens) can form a stable, covalent bond with a larger protein or peptide, resulting in their immunogenicity. The “hapten/prohapten” theory is one of three well-established models involving the human leukocyte antigen (HLA) in T cell-mediated drug hypersensitivity. The other two existing concepts are the “p-I” model (pharmacological interaction with immune receptors) and the “altered peptide repertoire model”, which is completed by a concept without HLA involvement, namely the “altered T-cell receptor repertoire” model [88,89]. With the exception of AGEP (HLA class unknown), the severe cutaneous adverse reactions (SCAR) Stevens-Johnson syndrome/toxic epidermal necrolysis (SJS/TEN) (class I), drug reaction with eosinophilia and systemic symptoms (DRESS) (class I/II), and drug-induced hypersensitivity syndrome (DIHS/HSS) (class I/II) are associated with HLA class I and/or II [88].

### 9.1. AGEP Is a Reversible Cutaneous Adverse Reaction

AGEP is characterized by the rapid development of numerous non-follicular sterile pustules in the epidermis, sometimes in response to the systemic application of drugs such as terbinafine, sertraline, and hydroxychloroquine [90,91,92]. The characteristic edematous erythema and sterile pustular eruption disappear and completely recover within 15 days of discontinuation of the drug or cessation of topical application, in contrast to other severe cutaneous adverse reactions, such as SJS/TEN, DRESS, and DIHS/HSS [88,93]. The precise cellular mechanism by which AGEP is triggered remains uncertain.

In AGEP-affected skin, keratinocytes exhibit an increased expression of the neutrophil-attracting chemokine IL-8 yet lack the expression of major histocompatibility complex (MHC) class II [87,88,89,91,94,95]. This indicates that instead of an HLA/MHC-associated immune response, the trigger of AGEP may involve an interaction of small molecules with cellular or subcellular structures, respectively, rather than proteins of the immune response. Repeated application can result in the cutaneous adverse effect of AGEP, but this does not necessarily occur [96]. This argues against IgE, B-cell, or T-cell involvement. One possible type of reversible interaction between small molecules and cellular structures or compartments is their lysosmotropism.

### 9.2. Lysosomotropism

Lysosomotropism is a frequently occurring biological characteristic of small molecules that leads to their accumulation in lysosomes, regardless of their chemical nature or mechanism of uptake [97]. Lysosomotropic compounds are weak organic bases (pKa > 6, lipophilic) that, uncharged, readily penetrate the lysosomal membrane, become protonated, and are subsequently trapped in the lysosomal lumen [64,98], reaching intra-lysosomal concentrations of up to 100 times higher than the cytosolic concentrations. Their accumulation leads to an increase in lysosomal pH from 4.5–5 to 6–6.5 [81]. This is reflected in the activity and substrate specificity of lysosomal enzymes [64,75,77,78,84].

### 9.3. Lysosomotropism of Drugs and AGEP

Topical application of lysosomotropic amitriptyline [64,99] in a 2% cream to healthy skin is typically well tolerated with hardly any or no adverse effects [100,101]. However, application to the skin of individuals with (UV–) light-sensitive skin, to skin prone to rash, itching, and AD, may cause severe cutaneous adverse reactions such as severe rash, pruritus, and papule formation (AGEP) [96,100]. Indeed, a number of lysosomotropic drugs, including the aforementioned sertraline and terbinafine, as well as loratadine and its lysosomotropic metabolite desloratadine, have been demonstrated to induce the sometimes severe cutaneous adverse effects, including rash, itching, erythema, maculopapular rash, pruritus, urticaria, and photosensitivity (AGEP) [88,91,98,100,102,103,104]. It is obvious that lysosomotropism plays a pivotal role in the triggering of AGEP. However, since lysosomotropism is an invariable molecular feature, the spontaneous and inconsistently reproducible onset of AGEP cannot be entirely explained. In AD-prone skin, the onset of AGEP appears to be associated with a decrease in very long-chain and ultra-long-chain ceramides and an increase in long-chain ceramides [5,14,96], suggesting limited ELOVL activity. As the ELOVL elongation of fatty acids fluctuates due to coupling to intracellular NADPH, this may be the variable key factor sought.

### 9.4. AGEP in the Compartmentalized Model

**Figure 4:**
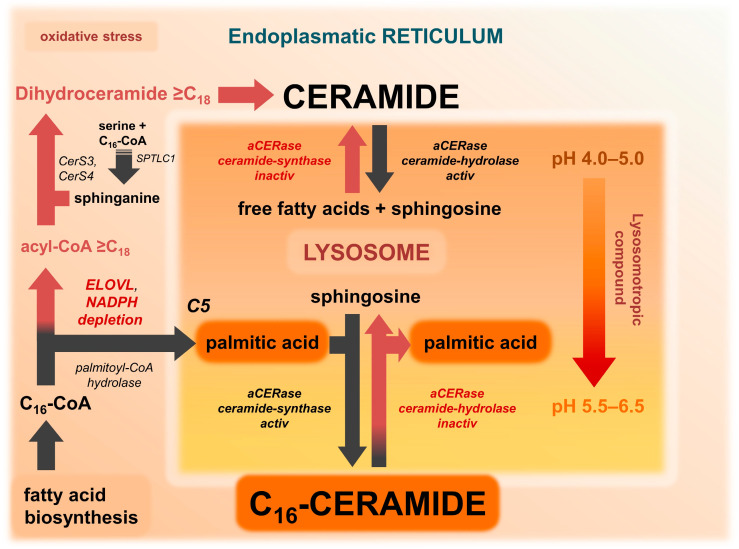
Lysosomotropism and impaired ELOVL6 initiated fatty acid elongation. The synthesis of pro-apoptotic and in AD strikingly increased C_16_-ceramide is dependent on two key factors: lysosomal pH (type of aCERase activity) and NADPH availability (activity of ELOVL6). Oxidative stress-induced depletion of NADPH, and subsequent termination of ELOVL6-initiated fatty acid elongation provide the optimal conditions for the formation of C_16_-ceramide in cells when a lysosomotropic compound is present (black arrows). C5 (condition 5)—lysosomotropism of compounds/drugs (lysosomal pH elevated) and impaired ELOVL6 elongation. Condition 5 can be considered as a variant of condition 3 (C3) in Figure 3, where the trigger of the pH increase is a lysosomotropic compound/drug (Table 1).

**Figure 5:**
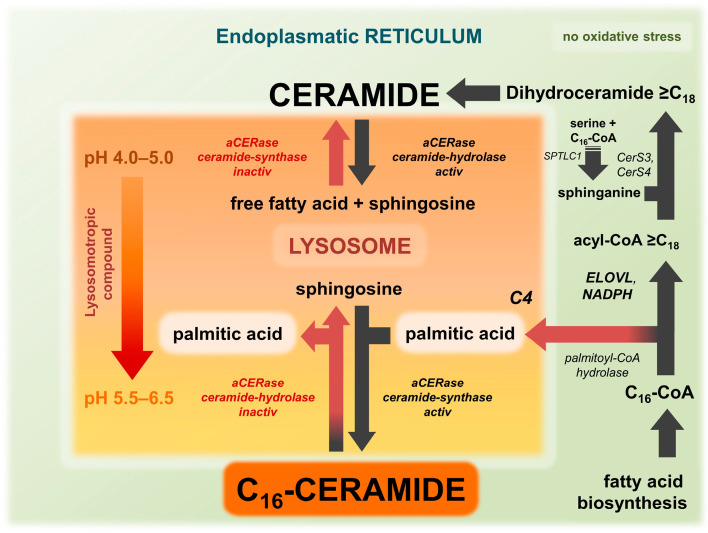
Lysosomotropism and active ELOVL6 initiated fatty acid elongation. The synthesis of the C_16_-ceramide in the presence of lysosomotropic compounds/drugs can be inhibited by stabilizing the ELOVL6 initiated fatty acid elongation. Despite an increase in lysosomal pH, the quantity of palmitic acid available for the synthesis of C_16_-ceramid is minimal. C4 (condition 4)—lysosomotropism of compounds/drugs (lysosomal pH elevated) and active ELOVL6 elongation. Condition 4 can be considered a variant of condition 1 (C1) in Figure 3), wherein the trigger of the pH increase is a lysosomotropic compound/drug (Table 1). Given that the ELOVL6-initiated fatty acid elongation is not terminated, the accumulation of ultra-long-chain and very long-chain ceramides will occur in lysosomes at pH above 5.5 similar to condition 1 (inhibition of aCERase ceramide hydrolase activity. Black arrows indicate active pathways, while red arrows indicate inactive pathways.

As previously discussed, the compartmentalized model of ceramide synthesis provides a reasonable explanation for the alterations in the levels of long-chain and very long-chain ceramides observed in lesional skin in AD. However, the potential of the model extends beyond this aspect. With minor modifications, the model (Figure 3) can be adapted to the presence of lysosomotropic compounds (Figure 4), thus explaining this apparently random cutaneous response to such drugs. The underlying hypothesis is that in AGEP, C_16_-ceramide and/or C_18_-ceramide are elevated in the skin and are therefore involved in the signaling pathway. In order to differentiate from other AGEP-inducing compounds that are not lysosomotropic and whose triggering mechanism is not well understood, the presence of lysosomotropic compounds is considered as separate conditions 5 (ELOVL6 elongation impaired) (Figure 4) and 4 (ELOVL6 elongation active) (Figure 5) and within the compartmentalized model (Figure 3 and Table 1). Non-lysosomotropic small molecules, including beta-lactam antibiotics, quinolones, and diltiazem, which have been demonstrated to induce AGEP [88], can also be included in the compartmentalized model. These molecules are likely to interact with intracellular processes that affect and increase the lysosomal pH.

### 9.5. Condition 5: Lysosomotropism of Drugs and ELOVL6 Elongation Impaired

According to the compartmentalized model of ceramide synthesis, C_16_-ceramide formation requires both an elevated lysosomal pH and an impaired ELOVL6-initiated fatty acid elongation (condition 3) (Figure 3). In the absence of an elevated pH, impairment of ELOVL elongation has minimal effects due to the lack of revaCERase/ceramide synthase activity (condition 2). In these conditions, the addition of a lysosomotropic drug, whether by topical application or by oral ingestion and subsequent accumulation in the skin, will increase the pH in the lysosome of keratinocytes and force the subsequent activation of revaCERase. Consequently, C_16_-ceramide is synthesized while concurrently less ceramide is hydrolyzed due to lack of aCERase activity (condition 5) (Figure 4). Condition 5 can be considered as a variant of condition 3, where the trigger of the pH increase is well known: lysosomotropic xenobiotics (Table 1).

### 9.6. Condition 4: Lysosomotropism of Drugs and ELOVL6 Elongation Active

Lysosomotropism of drugs or xenobiotics represents an (experimental) tool that is employed to trigger condition 1 (elevated pH with intact ELOVL elongation). Condition 4 can be considered as a special case of condition 1, where the trigger is very well characterized. In the presence of active ELOVL elongation and simultaneous inactivation of aCERase due to elevated pH, accumulation of very long-chain and ultra-long-chain ceramides occurs as previously described [64]. Systemic or topical application of lysosomotropic drugs (e.g., 2% amitriptyline) [101] is generally well tolerated in the presence of an intact ELOVL elongation (Figure 3 and Figure 5) [96,100]. Therefore, achieving condition 4 is the goal of therapy when AGEP occurs or stabilizing condition 4 as part of prophylaxis for skin prone to AD, for example.

### 9.7. AGEP—When Impaired ELOVL Fatty Acid Elongation Is Associated with Lysosomotropism

Drugs that are known to induce AGEP include several lysosomotropic compounds, such as terbinafine, sertraline, and hydroxychloroquine [91,92,103]. These findings suggest that AGEP is a lysosome-related phenomenon. In light of the model of compartmentalized ceramide synthesis, AGEP can be postulated as a transition from condition 2 (normal lysosomal pH and impaired ELOVL6-initiated fatty acid elongation) to condition 5 (elevated lysosomal pH and ELOVL6 initiated fatty acid elongation impaired) forced by lysosomotropic drugs or other lysosomotropic compounds (Figure 3 and Figure 4 and Table 1). The delay in the onset of clinical symptoms is most likely due to the duration of histologic remodeling.

### 9.8. AGEP and Other Compounds That Cause a Lysosomal Proton Gradient Depolarization

While small molecule lysosomotropism is the most prominent way to increase lysosomal pH, other less well-known methods to induce lysosomal pH increase include blockade of either the lysosomal proton pump, drug-induced impairment of the transmembrane proton gradient (e.g., by metformin [105,106]), or increased free radical production by the inducing drug. This broader characterization of candidate compounds allows the compartmentalized model to cover more than just lysosomotropic compounds as inducers of AGEP. The extent to which the well-known AGEP inducers, beta-lactam antibiotics, and quinolones [88] are covered by the model has not yet been clarified.

## 10. Impairment of ELOVL6 Elongation and Related Adverse Effects of Topical Drugs and Cosmetics

Insufficiently quenched ROS stress at the ER depletes NAD(P)H, increasing the likelihood of impaired ELOVL6-initiated fatty acid elongation and the lysosomal proton pumps. Given these skin conditions, the application of topical formulations such as creams, ointments, or gels containing significant amounts of lysosomotropic compounds, free palmitic acid, or its derivatives (e.g., esters) carries the risk of causing undesirable cutaneous adverse effects. When considering the topical application or oral application of a drug, it is important to pay attention to the possible or proven lysosomotropism of the drug, especially if the drug is known to accumulate in the skin. A concomitant unrecognized impaired ELOVL6 fatty acid elongation poses a greater risk for the occurrence of AGEP, according to the model (Figure 3 and Figure 4). These high-risk drugs include prominent and commonly used over-the-counter (OTC) drugs such as the antihistamine dimetindene or the antifungal terbinafine, as well as prescription creams containing amitriptyline (2%) or ambroxol for neuropathic pain [98,101,107,108]. The hypothesis and mechanism presented in Figure 4 have been inadvertently confirmed by the application of a 0.03% amitriptyline cream to a recently restored lesional skin area in AD [96].

**Figure 6:**
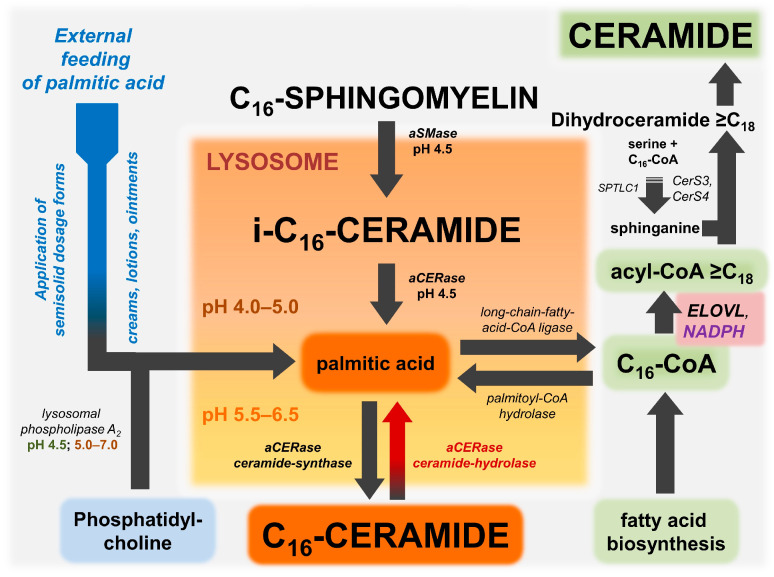
Palmitic acid: A key molecule in lipid metabolism that is altered in AD [14,15]. The occurrence and metabolism of palmitic acid are dependent on lysosomal pH and the presence of NADPH. Potential sources include phosphatidylcholine, C_16_-ceramide, and C_16_-CoA from fatty acid biosynthesis and external supply. Intermediate C_16_-ceramide (i-C_16_-ceramide) is released from, for example, SM and immediately undergoes hydrolysis to yield sphingosine and palmitic acid.

Furthermore, an excess supply of palmitic acid, whether through creams or ointments for basic skin care in AD or in cosmetics where palmitic acid is approved as a skin conditioner (emollient) or as an emulsifying surfactant [109], can promote the synthesis of C_16_-ceramide in keratinocytes with elevated lysosomal pH (e.g., in AD or after radiotherapy) [23,76]. When the trans-lysosomal proton gradient decreases, resulting in an increase in pH within the lysosomes of keratinocytes in lesional skin in AD [14,76], any extra palmitic acid from creams or ointments that cannot be converted to palmitic acid-CoA is made available to the lysosomal revaCERase for synthesis with sphingosine to form C_16_-ceramide (condition 3 and condition 5). This can result in an unanticipated and, above all, unintended exacerbation of the skin condition rather than the intended improvement (Figure 4 and Figure 6).

## 11. Increasing the Tolerance to Lysosomotropic Drugs in AD and Basic Prophylaxis of AGEP

Drug tolerance and safety to AGEP can be defined as the non-appearance of AGEP during drug application. According to the compartmentalized model of ceramide synthesis, this tolerance and safety is dependent on the status of ELOVL elongation of ultra-long and very long-chain fatty acids and a minimum of free palmitic acid in the lysosome (Figure 4 and Figure 5). If ELOVL fatty acid elongation remains unimpaired (condition 4), the release of palmitic acid by hydrolysis from excess C_16_-acyl-CoA can be minimized, and the desired low level of free palmitic acid in the lysosome is achieved. Consequently, the restoration and stabilization of ELOVL6-initiated fatty acid elongation in keratinocytes (transition from condition 5 to condition 4) (Figure 5 and Table 1) represents a key objective in the management and control of cutaneous adverse effects of lysosomotropic drugs and drug-induced AGEP. With non-lysosomotropic compounds such as metformin, which increase the pH in the lysosome by a different mechanism, the objective is to achieve a transition from condition 3 to condition 1 (Table 1). Elevated lysosomal pH, whether caused by lysosmotropic compounds or diminished proton gradient (e.g., by compounds or NADH/ATP depletion), can be well tolerated if as little free palmitic acid as possible is present in the lysosome for the synthesis of C_16_-ceramide by revaCERase.

### 11.1. Linoleic Acid Can Stabilize ELOVL6 Fatty Acid Elongation and Increase Tolerance to AGEP

Minimizing the stress at the ER is of paramount importance to prevent a combination of elevated lysosomal pH by lysosomotropic compounds and impaired ELOVL6 fatty acid elongation, which leads to lysosomal C_16_-ceramide synthesis (as shown in Figure 3, Figure 4 and Figure 5, and Table 1). Naturally occurring polyunsaturated fatty acids (PUFA) are potent endogenous peroxisome proliferator-activated receptor (PPAR) ligands [110], and among them, linoleic acid in particular, is considered to be an excellent ROS quencher, a peroxisome proliferator-activated receptor gamma (PPARG) ligand and activator [111], an inducer of PPARG expression [112], and an activator of fatty acid oxidation. The fact that PPARG also increases the expression of ELOVL4 and ELOVL7, as well as CerS3 [21], all of which are involved in the endogenous synthesis of ultra-long-chain and very long-chain ceramides, suggests that linoleic acid is a promising compound that may be able to improve tolerance to lysosmotropic drugs and thus control the predisposition to develop AGEP during application. Consequently, linoleic acid may be capable of achieving the requisite stabilization of ELOVL6-initiated fatty acid elongation to tolerate an elevated lysosomal pH (transition to or stabilization of condition 4) (Figure 5). Stabilization of ELOVL fatty acid elongation by linoleic acid can effectively prevent the hydrolysis of excess C_16_-CoA from the fatty acid synthesis to free palmitic acid.

### 11.2. Tolerance to AGEP and Elimination of the Cutaneous Adverse Effect

Topical application of PPARG activators, such as linoleic acid, as previously described, may be a convenient and simple method to normalize NAD(P)H and ATP levels and stabilize condition 4 or condition 1 within keratinocytes (Figure 3 and Figure 5). This is particularly applicable if the triggering compound is lysosomotropic or the trigger of the existing lysosomal pH increase is still unknown. Pretreatment of the skin with linoleic acid-containing creams enables the topical application of lysosomotropic drugs such as amitriptyline (e.g., for pain relief) to lesional skin and skin prone to AD [96,101] and increases drug tolerance to lysosomotropic (active) compounds/drugs, including tolerance under more challenging conditions such as ROS-inducing intense UV radiation. Conversely, local application of linoleic acid in a topical formulation may alleviate AGEP, as demonstrated by the combination of amitriptyline and linoleic acid on AD-prone skin [96]. As linoleic acid is currently listed in the European Cosmetic Database (CosIng) as a skin conditioner (emollient) [109], its addition to creams is straightforward and the supplement of choice. The targeted use of linoleic acid as a stabilizer of the fatty acid elongation initiated by ELOVL6 is situated within the legal gray area between cosmetics and pharmaceuticals. This is due to the fact that the desired effect is a pharmacological effect of linoleic acid rather than the surface effect of an emollient.

## 12. Basic AD Therapy beyond Emollient and Moisturizer Recommendations

Effective treatment and maintenance (baseline) strategies for AD are characterized by the achievement of satisfactory remission or control of signs and symptoms and can be applied for extended periods of time if necessary. In order to maintain healthy skin without lesion recurrence, it is necessary to provide adequate skin care for the affected areas, as well as an effective prophylaxis against lesion recurrence. The most recent guidelines from dermatologic societies provide recommendations for treatment, prophylaxis, and emollients [6,113].

### 12.1. Current Basic Therapies, Emollients, and Moisturizers

The recently published guidelines on recommended basic therapies for AD focus on the delivery of (synthetic) lipids to the upper epidermis to passively restore the permeable skin barrier, with occludents to trap moisture in the upper layer of the skin to reduce TEWL. Recommended emollients typically include a moisturizer or humectant, such as urea or glycerol, to promote stratum corneum hydration, and occludents to reduce evaporation, such as lipids or liquid paraffin, white soft paraffin, and petrolatum [6,113,114]. Many of these formulations additionally contain ceramide 1 (EOP), ceramide 3 (NP), and ceramide 6 (AP) with a phytosphingosine [P] backbone [25] (Figure 1). They are designed to passively compensate for the lack of ultra-long-chain and very long-chain ceramides by substitution in order to restore the impermeable skin barrier. Given that EOS and EOH, but not EOP ceramides, are significantly decreased in lesional skin [14,17] and that different backbones directly affect the structure and density of the lipid lamellae formed [27,37,115], it is questionable whether the desired stabilizing effect will be achieved by the external addition of ceramide 1, ceramide 3, and ceramide 6.

### 12.2. Recommended Basic Therapy and ELOLVL Fatty Acid Elongation Impairment

Clinical trials of formulations containing ceramide surrogates have demonstrated improved skin hydration and reduced skin dryness. However, their effect on TEWL has not been investigated [24]. Therefore, it is challenging to assess whether the effect on the skin barrier extends beyond the demonstrated short-term benefit. In accordance with the guidelines, emollients containing additives such as flavonoids (e.g., licochalcone A), saponins, and riboflavins derived from protein-free oat plant extracts or organic compounds, such as ceresin, acting as occluders, are recommended as basic therapy and prophylaxis. These emollients are referred to as “emollients plus” because they exhibit an effect beyond their intrinsic, purely physical effect. Furthermore, they are also acting as putative active compounds without yet having to be classified as pharmaceuticals [6]. However, according to the model, no significant stabilizing or restoring effect on the ELOVL fatty acid elongation can be expected from the basic therapy, as apart from tocopherol acetate or butylated hydroxytoluene (BHT) as antioxidants to stabilize the formulation, no suitable and sufficiently effective compounds are included in the cream bases. It is, therefore, unlikely that the desired return of keratinocytes from condition 3 (AD) to condition 1, or ideally condition 0 (Figure 3), will occur as a result of emollients plus in topical formulations. Instead, this may represent a spontaneous remission. Without providing an adequate supply of intracellular NAD(P)H and ATP, neither physiological lysosomal pH nor ELOVL6-initiated fatty acid elongation can be restored, and subsequent and permanent stabilization of the ceramide metabolism in keratinocytes can be secured.

### 12.3. Linoleic Acid Can Recover Lesional Skin and Increases the Tolerance to AD

In the compartmentalized model of ceramide synthesis, AD (condition 3) and AGEP (condition 5) differ only in the trigger for the increase in lysosomal pH. The increase in pH in AGEP is reversible simply by stopping the triggering compound, whereas in AD, a specific intervention is required to lower the lysosomal pH. Given that a combination of unquenched ROS and reduced NAD(P)H and ATP levels is most likely to be present in keratinocytes in AD, the depletion of intracellular NAD(P)H and ATP stresses the ER, impairs both lysosomal proton pumps, and results in elevated lysosomal pH due to a lack of availability of the energy sources ATP (V-ATPase) and NADH (lysosomal RedOx chain) [70,72]. Experimental inhibition of fatty acid oxidation has been demonstrated to decrease NAD(P)H production and increase free ROS, which leads to ATP depletion [54]. Furthermore, stress via γ-irradiation has been shown to elicit the well-known C_16_-ceramide synthesis and subsequent cell death [23]. To compensate for such deficits, augmented mitochondrial fatty acid oxidation, along with the oxidative branch of the pentose phosphate pathway [116], the two major sources of NADPH, may provide more NADPH to counteract existing oxidative stress and prevent ATP loss.

As with AGEP, the compartmentalized model suggests that the overriding stabilization of ELOVL6-induced fatty acid elongation is a viable solution to remedy the deficiency. This is evident from Figure 3 and Figure 5, which reveal that an elevated lysosomal pH can be well tolerated if as little free palmitic acid as possible is present in the lysosome for C_16_-ceramide synthesis (transition from condition 3 to condition 1). Nevertheless, achieving the optimal condition 0 is not necessary. Once again, the topical application of PPARG activators such as linoleic acid, as previously described with AGEP, may prove to be a convenient tool to normalize NAD(P)H and ATP levels within keratinocytes and subsequently stabilize ELOVL6-initiated fatty acid elongation, including resistance to more challenging conditions such as ROS-inducing intense UV radiation.

## 13. Other Applications of the Model in Lipid Metabolism-Related Diseases

The compartmentalized model of ceramide synthesis (Figure 3) provides a more comprehensive explanation for the altered ceramide composition and levels observed in AD. Furthermore, the model can be utilized to understand additional phenomena that can be attributed to a disorder in ceramide/lipid metabolism. A prominent example is the common polymorphic light eruption (PMLE/PLE) or Mallorca acne.

PLE and AGEP are both recurrent skin disorders that share common features, including rash; non-scarring, pruritic, erythematous papules; papulovesicles; vesicles; or plaques with delayed onset. These features manifest following the application of a drug (AGEP) or exposure to intense UV radiation, such as sunlight (PLE) (Table 2). In both AGEP and PLE, the affected skin areas resolve completely without scarring when the trigger is discontinued [91,93,95,117,118,119]. PLE is induced by intense UV radiation and inadequately quenched UV-generated ROS, leading initially to NADPH depletion and impairment of ELOVL fatty acid elongation (condition 2). Further depletion of NAD(P)H/ATP leads to impairment of the lysosomal proton pumps and subsequent elevation of lysosomal pH (condition 3), which fosters the well-known synthesis of C_16_-ceramide (or C_18_-ceramide) by revaCERase to occur and can trigger the subsequent formation of pruritic pustules. Similarly, it is conceivable that the inadequately quenched UV-generated ROS facilitate the synthesis of compounds within the skin that effectively diminish or nearly abolish the lysosomal proton gradient, a mechanism of action comparable to that observed with metformin [105,106]. This proposed mechanism of action may explain the etiology of topical phototoxicity (photosensitization, phytophotodermatitis) of the psoralens 5-methoxypsoralen and 8-methoxypsoralen in plants and fruits [119] or some cutaneous side effects (erythema, rash, and blistering) of radiotherapy [23,120,121].

In the event of PLE or phototoxicity, the hypothesis and the compartmentalized model of ceramide synthesis (Figure 3) suggest the application of the PPARG activator linoleic acid as a potential remedy for the aforementioned symptoms, including pruritic rash and erythema. Moreover, this approach is recommended as part of prophylaxis and effective baseline therapy of PLE. This model-based strategy surpasses the currently available, rather unspecific, and often ineffective recommendations, such as sun avoidance, sunscreen, topical corticosteroids, and systemic corticosteroids in severe cases; the oral prophylaxis with β-carotene, nicotinamide ω-3 polyunsaturated fatty acids; and the topical prophylaxis with antioxidants such as α-glycosylrutin, tocopherol acetate and ferulic acid [118,122].

## 14. Conclusions

The existing models have localized ceramide synthesis exclusively to the ER and neglected the dual nature of aCERase [38,48,60,123]. However, the formation of specific C_16_-ceramide and 2-hydroxy C_16_-ceramide during apoptosis cannot be consistently explained by these models. The compartmentalization of ceramide synthesis (Figure 3) provides a more accurate explanation of the changes that occur in AD. The model proposes that the disease-associated ceramides, C_16_-ceramide and 2-hydroxy C_16_-ceramide, are synthesized in the lysosome. In contrast, the vital very long-chain and ultra-long-chain ceramides, which contribute to the lipid barrier of the skin, are synthesized via de novo synthesis at the ER. This compartmentalization elucidates the disparate responses of the two groups of ceramides in AD, as the two synthesis pathways permit (cutaneous) cells to respond in disparate ways to alterations in NAD(P)H levels. The overall status of ceramide synthesis for all subgroups is contingent on the availability of NAD(P)H and ATP.

The model’s key parameters, including lysosomal pH; NAD(P)H and ATP availability; and ELOVL fatty acid elongation activity, provide an insight into the overall status of the ceramide metabolism and of the preferentially synthesized and enriched ceramides in cells. Mitochondrial fatty acid oxidation is a key cellular process for supplying the skin with NAD(P)H and ATP and for the subsequent full differentiation of keratinocytes to form a robust skin barrier. At the enzymatic level, the lysosomal aCERase, which exhibits a pH-dependent long-chain ceramide synthase activity (revaCERase, optimum pH 5.5–6.5) within the lysosome, and the NADPH-dependent step-in enzyme ELOVL6 for the very long-chain fatty acid elongation are the key enzymes of the model. Their activity is dependent on the key parameters lysosomal pH, NAD(P)H, and ATP. The exceptional dual nature of aCERase, which enables selective pH-controlled synthesis of long-chain C_16_-ceramide in the lysosome and is provided with substrate palmitic acid by impaired ELOVL fatty acid elongation, thereby being promoted, represents a unique feature of the compartmentalized model of ceramide synthesis in comparison to existing models. The impairment of ELOVL fatty acid elongation at the ER, in conjunction with a simultaneous increase in lysosomal pH, is regarded as the trigger for the specific long-chain ceramide biosynthesis and accumulation in the lysosome. Furthermore, the compartmentalized model of ceramide synthesis (Figure 3) is not confined to AD. This model is also applicable to other lipid metabolism-related phenomena, such as AGEP (with a triggering drug or compound that directly modifies lysosomal pH) or PLE (with other triggers than drugs or compounds that directly modify lysosomal pH).

The hypothesis suggests that in the event of NAD(P)H and ATP deficiency in AD, AGEP, or PLE, the administration of PPARG activators such as linoleic acid may serve to address the underlying issue and result in a remission of symptoms or provide effective prophylaxis. This strategy of stabilizing the endogenous synthesis of very long-chain and ultra-long-chain fatty acids and ceramides in AD pursued with the concept is considered to be a superior approach to the application of moisturizers, occludents, and “emollients plus” in prophylaxis [6], or the compensation of ceramide [S] deficiency by substitution with ceramide 1 (EOP), ceramide 3 (NP), and ceramide 6 (AP), each containing the phytosphingosine [P] backbone.

In conclusion, the compartmentalized model provides an approach to a more comprehensive understanding of the etiology of AD and other lipid metabolism-related diseases at the level of lipids and ceramide metabolism. This understanding can then be utilized to develop new therapies and prophylactic methods that lead to complete remission or predictable disease progression.

Lysosomotropic compounds exert a lysosome-driven modulating effect on the level of gene expression, lysosomal protein maturation, and subsequent release of chemokines, cytokines, interleukins, enzymes, and receptors including CXCL3, CXCL10, PTX3, PTGS2 (COX2), IL-6, and TNF-alpha [64,124,125,126,127]. While lysosomotropic drugs are significantly beneficial in systemic viral and bacterial infections with regard to disease severity, outcome, and survival when the route of infection is via phagocytosis and the endolysosomal pathway [126,128,129,130,131], their impact on healthy skin, on Staphylococcus aureus-associated colonization present on lesional skin in AD [132], and the local dermal immune system remains to be fully elucidated and will be discussed in detail elsewhere.

The compounds and preparations described here for maintaining ELOVL fatty acid elongation have not yet been optimized to their full potential. Even now, the currently known compounds, such as the approved PPARG activator linoleic acid, enable simple prophylaxis and treatment of AGEP and PLE via the use of magistral preparation or commercially available creams. The presented concept is intended to complement and support current therapies with highly potent corticosteroids or antibodies in moderate to severe cases of AD. However, at present, it is not anticipated to be a complete replacement for these existing treatments.

## Figures and Tables

**Table 1:** Expected alterations in long-chain ceramide (C_16_), very long-chain ceramides, and ultra-long-chain ceramides (>C_20_/>C_26_) depending on lysosomal pH and ELOVL fatty acid elongation (dys)function, derived from the compartmentalized ceramide metabolism model (Figure 3): (o) unchanged to hardly changed; (+) increased; (++) strongly increased; (-) decreased; (--) strongly decreased. Lyosomotropic compound/drug: (+) present; (-) absent. A numerical value (0–5) is assigned to each combination of pH and ELOVL status (condition). Condition 0 corresponds to the physiological state of the lysosome; lysosomotropic compounds (drugs) that may cause adverse effects, such as AGEP, are considered in conditions 4 and 5. The physiological lysosomal pH range of 4.0–5.0 (black) represents the activity optimum for aCERase, while the elevated pH range of 5.5–6.5 (red) corresponds to revaCERase and lysosomal C_16_-ceramide synthesis [77,78]. For simplicity, either aCERase or revaCERase activity is assumed. However, the transition in the cell is smooth with a mixture of both activities. Lysosmotropic compounds/drugs can elevate the pH to values between 6.0 and 6.5 [81].

Condition	Lysosomal pH	ELOVL Elongation	Long-Chain Ceramide (C_16_)	Very Long-Chain and Ultra-Long-Chain Ceramide (>C_20_/>C_26_)	Lysosmotropic Compound/Drug
0	4.0–5.0 ^#^	active	o	o	-
1	5.5–6.5 *	active	o	+	-
2	4.0–5.0 ^#^	impaired	o	o/-	-
3	5.5–6.5 *	impaired	+/++	--	-
4	5.5–6.5 *	active	o	+	+
5	5.5–6.5 *	impaired	+/++	--	+

* revaCERase (aCERase ceramide synthase activity) is active, ^#^ aCERase (ceramide hydrolase) is active.

**Table 2:** Comparison of AGEP, PLE, and AD. Expected changes in long-chain ceramide (C_16_) and very long-chain/ultra-long-chain ceramides (>C_20_/>C_26_) depending on pH and dysfunction of ELOVL fatty acid elongation in AGEP, PLE, and AD, derived from the model of compartmentalized ceramide metabolism (Figure 3): (o) unchanged to hardly changed; (+) increased; (++) strongly increased; (-) decreased; (--) strongly decreased; (*) estimated. Lyosomotropic compound/drug: (+) present; (-) absent. Healthy skin, skin prone to AD, AD, AGEP, and PLE are assigned to each combination of pH and ELOLVL status. Physiological pH 4.0–5.0 (black) defines the activity window for aCERase (lysosomal ceramide hydrolase), elevated pH 5.5–6.5 (red) for revaCERase (lysosomal ceramide synthase) and lysosomal C_16_-ceramide synthesis [77,78]. Lysosmotropic compounds/drugs can increase the pH to values of 6.0–6.5 [81]. (*) Lysosomal pH is estimated according to the model; no experimental data are available except for AD (^#^) [76]. Lysosomal pH determines the ratio of revaCERase to aCERase activity and the accumulation of C_16_-ceramide. The prevalent type of aCERase activity: (H) ceramide hydrolase (aCERase); (S) long-chain ceramide synthase (revaCERase).

	Lysosomal pH	ELOVL Elongation	Long-Chain Ceramide (C_16_)	Very Long-Chain and Ultra-Long-Chain Ceramide (>C_20_/>C_26_)	Lysosmotropic Compound/ Drug	Type of aCERase Activity
Healthy skin	4.0–5.0	active	o	o		H
Skin prone to AD	4.0–5.5 *	impaired	o/+	-/o/+	-	H
AD	5.5–6.5 ^#^	impaired	+/++	-/--	-	S
AGEP	5.5–6.5 *	impaired	+/++ *	-/-- *	+	S
Skin prone to PLE	4.0–5.5 *	impaired	o/+ *	-/o/+ *	-	H
PLE	5.5–6.5 *	impaired	+/++ *	-/-- *	-	S

## Data Availability

Not applicable.

## Abbreviations

aCERase	Acid ceramidase
AD	Atopic dermatitis
AGEP	Acute generalized exanthematous pustulosis
aSMase	Acid sphingomyelinase
CerS	Ceramide synthase
DIHS/HSS	Drug-induced hypersensitivity syndrome
DRESS	Drug reaction with eosinophilia and systemic symptoms
ELOVL	Very long-chain-3-oxoacyl-CoA synthase
FAS	Fatty acid synthase
HLA	Human leukocyte antigen
INCI	International Nomenclature of Cosmetic Ingredients
LPLA2	Lysosomal phospholipase A2
MHC	Major histocompatibility complex
nCERase	Neutral ceramidase
nSMase	Neutral sphingomyelinase
PMLE/PLE	Polymorphous light eruption
PPARG	Peroxisome proliferator-activated receptor gamma
PUFA	Polyunsaturated fatty acid
OTC	Over-the-counter (drugs)
revaCERase	Reverse acid ceramide synthase/acid ceramidase synthase activity
ROS	Reactive oxygen species
SCAR	Severe cutaneous adverse reactions
SJS/TEN	Stevens-Johnson syndrome/toxic epidermal necrolysis
TEWL	Transepidermal water loss
V-ATPase	Vacuolar type (H+)-ATPase.
**Nomenclature of ceramides and fatty acids:**	
Long-chain	C_14_–C_18_/C_20_
Very long-chain	C_20_–C_26_
Ultra-long-chain	>C_26_.
**Ceramide backbones and fatty acid residue:**	
[dS]	Dihydrosphingosine (sphinganine)
[S]	Sphingosine
[P]	Phytosphingosine
[H]	6-hydroxy-sphingosine
[N]	Non-hydroxy fatty acid
[A]	2-hydroxy fatty acid
[EO]	Esterified ω-hydroxy fatty acid.
